# Sex Differences in Behavioral Dyscontrol: Role in Drug Addiction and Novel Treatments

**DOI:** 10.3389/fpsyt.2015.00175

**Published:** 2016-02-08

**Authors:** Marilyn E. Carroll, John R. Smethells

**Affiliations:** ^1^Department of Psychiatry, University of Minnesota, Minneapolis, MN, USA; ^2^Program in PharmacoNeuroImmunology, Department of Psychiatry, University of Minnesota, Minneapolis, MN, USA

**Keywords:** animal models, behavioral dyscontrol, drug addiction, food addiction, impulsivity, sweet intake, sex differences, novel treatments

## Abstract

The purpose of this review is to discuss recent findings related to sex differences in behavioral dyscontrol that lead to drug addiction, and clinical implications for humans are discussed. This review includes research conducted in animals and humans that reveals fundamental aspects of behavioral dyscontrol. The importance of sex differences in aspects of behavioral dyscontrol, such as impulsivity and compulsivity, is discussed as major determinants of drug addiction. Behavioral dyscontrol during adolescence is also an important consideration, as this is the time of onset for drug addiction. These vulnerability factors additively increase drug-abuse vulnerability, and they are integral aspects of addiction that covary and interact with sex differences. Sex differences in treatments for drug addiction are also reviewed in terms of their ability to modify the behavioral dyscontrol that underlies addictive behavior. Customized treatments to reduce behavioral dyscontrol are discussed, such as (1) using natural consequences such as non-drug rewards (e.g., exercise) to maintain abstinence, or using punishment as a consequence for drug use, (2) targeting factors that underlie behavioral dyscontrol, such as impulsivity or anxiety, by repurposing medications to relieve these underlying conditions, and (3) combining two or more novel behavioral or pharmacological treatments to produce additive reductions in drug seeking. Recent published work has indicated that factors contributing to behavioral dyscontrol are an important target for advancing our knowledge on the etiology of drug abuse, intervening with the drug addiction process and developing novel treatments.

## Introduction

Addiction and related impulse control disorders have an estimated cost to society of 600 billion dollars per year (1). In humans, substance abuse varies with current drug availability trends and sex differences are reported (2), but the direction of those differences is not always consistent, as it varies with current trends in substance availability and cost (3). However, in recent years, women exceed men in the abuse of prescription drugs, men use more alcohol and stimulant drugs than women, and the use of nicotine has been more equal across sexes (4). By contrast, animal studies have revealed more consistent trends in drug-seeking behavior indicating that females are more likely to initiate and maintain drug-seeking behavior, and they have a better response to treatment than males. Sex differences in drug abuse, hormonal influences, and their implications for treatment have been extensively reviewed with regard to animal and human studies (3, 5–9). A recent finding that is important to our understanding of sex differences in drug abuse is that underlying aspects of addiction, such as compulsive and impulsive behavior (e.g., behavioral dyscontrol) are strong determinants of addiction, and differences in drug taking depend on several factors, such as type of drug, behavioral measures that are used, and sociocultural influences [e.g., Ref. (10)].

The present review focuses on elements of behavioral dyscontrol that increase vulnerability to drug abuse and add to sex differences to evaluate the importance of sex, hormonal conditions, as well as other individual differences, in developing treatments for drug addiction. In this review, findings from *laboratory animals* and *human research* will be discussed separately. We discuss major aspects of behavioral dyscontrol and how they interact with sex differences to contribute to vulnerability to drug addiction and receptivity to treatment. Behavioral dyscontrol is defined as maladaptive influences, behavior that an individual has difficulty stopping. It includes impulsivity, compulsive binge-like behavior, and it is particularly prevalent during adolescence (vs. adulthood), the time when drug addiction is most prevalent. In several reviews of factors that underlie drug addiction, these topics have emerged as the strongest contributors to addictive behavior, and there are interactions among them. The goal of this review is to bring the sex differences and behavioral dyscontrol literature together within the drug-abuse context to better understand critical vulnerability factors for drug addiction and how that knowledge may be useful in developing prevention and treatment strategies. Parallels are also drawn to other forms of addiction, such as food addiction (11–13), to illustrate that mechanisms of dyscontrol that underlie these addictive behaviors are similar. Thus, it may be instructive to target elements of dysregulation such as impulsive and compulsive behavior when developing strategies to treat drug addiction. For each of the following determinants of drug addiction, results from animal and human studies will be considered separately with respect to sex differences.

The study of sex differences in addiction has branched into several directions since our initial work of the late 1990s [see Ref. (14)]: the next section (Section 2) compares *sex differences* observed in *human and animal models* of drug abuse to understand how this factor affects addiction potential. Section 3 focuses on the *underlying processes of addiction*, such as *impulsivity that leads to drug seeking*. Thus, differences in addictive behavior in rats selected for high vs. low impulsivity (HiI vs. LoI) will be discussed and results extended to human drug addiction. Section 4 considers compulsive behavior that makes addictive behavior persist by comparing sex differences in *compulsive, binge-like characteristics of addiction* using selectively bred rat lines that were bred to binge drink a saccharin (SACC) solution (HiS) vs. rats that consume low to normal levels of SACC (LoS). These HiS and LoS rat lines are genetically predisposed to show high vs. low levels of drug seeking. These findings are discussed with respect to food addiction and its similarity to drug addiction. Section 5 compares sex differences during a *critical developmental* period (adolescents vs. adults) on measures of behavioral dyscontrol and drug addiction. Comparing different ages is a natural study in behavioral dyscontrol, as adolescence is the time when most humans express higher rates of impulsivity, compulsivity, and drug-seeking behavior compared with adults. Finally, Section 6 considers sex and individual differences in response to *treatment for behavioral dyscontrol and drug addiction*. Novel treatments are discussed, such as environmental enrichment, competing rewards/activities (e.g., exercise), and consequences (positive and negative). Pharmacological treatments are also discussed, such as medications to target underlying factors in addiction, such as impulsivity and anxiety. Self-sustaining treatments and customized treatments for addiction-prone and -resistant phenotypes are examined, as well as novel treatment combinations.

## Sex and Hormonal Influences on Drug Addiction

Sex differences in drug addiction is an area of research that has received increased attention since it began over 15 years ago, when the early studies were first reviewed (14), to the present when research in both sexes at all levels of animal research is recommended (15), and will be mandated by NIH (16). Sex differences in drug addiction have been discussed in recent reviews of animal and human studies (3, 5–8, 17). In non-human animals, the direction of the sex differences generally favors greater avidity for drugs in females than males, and this is mainly due to hormonal differences. However, sex differences in studies of drug addiction in humans are less clear, as they are influenced by both hormonal conditions and societal factors (3). A key factor in understanding sex differences in drug addiction is that in females estrogen increases drug-seeking and drug-rewarding effects, whereas progesterone (PRO) decreases drug-seeking behaviors to the levels of males. By contrast, male hormones (e.g., testosterone) have little influence on drug addiction. The importance of sex differences and hormonal conditions in addictive behaviors in animal and human research has been recently reviewed (3, 5, 7, 8, 17–20) and is summarized in separate sections below along with suggestions of areas where future work is needed.

### Laboratory Animals

Preclinical findings on vulnerability factors in drug abuse have been extensively reviewed in recent years [e.g., Ref. (5–7, 17, 18, 21–28)]. These reviews also indicate that sex differences occur in each phase of the addiction process ranging from initiation (acquisition) to maintenance, escalation, withdrawal, and relapse. In general, female animals exhibit greater vulnerability than males to drug-seeking behavior during most of these phases. An exception is that during drug withdrawal (26, 29–31), and when considering other negative or punishing effects of drugs (22, 23), males generally show more susceptibility than females to these negative drug-related effects. While sex differences in animal research occur across most phases of addiction, and they are consistent, the effects are modest in size. Observing a sex difference effect in drug addiction research is usually depend on the use of drug naive animals, low-to-moderate drug doses, and/or relatively demanding reinforcement schedules. Sex differences are not as likely to be found at high doses, or when access to the drug requires minimal effort, as ceiling effects occur.

However, there are relatively few reports of substantial sex differences in humans compared to the stronger sex-specific effects found in animals, which may be explained by environmental conditions. In experimentally naive animals, background conditions and experimental environments are minimal and typically the same for all animals, focusing on one variable, sex differences or hormonal conditions, with only minimal non-drug rewards available in the drug-taking environment (e.g., food, water). Thus, sex differences in drug seeking are more likely to emerge in animals (vs. humans) and be attributed to the rewarding effects of the drug, as the drug is a highly salient commodity. By contrast, in human studies, participants are not drug naive, there is environmental diversity (vs. uniformity), and historical factors as well as concurrently available of competing rewards can also influence self-reports of the rewarding effects of abused drugs. Another difference is that drug abuse is measured by actual drug intake in animals, whereas it is measured by self-report and choice or hypothetical choice in humans.

### Humans

Sex differences in drug effects are reported in humans, and they are similar to those found in animals; females generally exceed males in drug use, although to different degrees with different types of drugs (5). For example, in recent years, women were more likely than men to use prescription drugs, such as sedatives, tranquilizers, and opioids for pain, whereas men were more likely to use illicit drugs (32). Overall, in humans, females are more avid drug seekers than males with regard to several drugs of abuse, such as alcohol (33), amphetamine (34), cannabinoids (19), cocaine (14, 20), nicotine (35), opioids (36), and phencyclidine (37). By contrast, as reported in studies with rats and monkeys, males generally show more susceptibility than females to negative drug effects, such as drug withdrawal (38).

Sex differences in drug addiction in humans are strongly influenced by biological conditions such as estrogen and PRO levels (39) and the PRO (P) to estrogen (E) ratio (P/E) (40) during different phases of the menstrual cycle that has been suggested as an index of hormonal status in humans (40). However, sex differences in drug addiction are also determined by sociological as well as biological factors (10, 17). For example, when considering sex differences in human alcohol and opioid use in the 1700s, and comparing that to alcohol, opioid use in the late 1800s to early 1900s, it is clear that as social norms and legal policy changed, drug-abuse patterns have shifted in women and men over time (17). Kornetsky (10) illustrated that due to cultural changes and changes in job opportunities, family structure, and other social factors, alcohol and opioid abuse were more common in women than men in the 1800s, but in the 1900s, men exceeded women on use of most drugs, and those patterns shifted, depending on the drug, in the late 1990s to the present.

### Current Research on Sex Differences in Addiction

The study of sex differences in drug addiction did not begin in earnest until about 1998 [see Ref. (14)]. In recent years, the study of sex differences (41) and the impact of ovarian hormones (42) have expanded exponentially and taken on several new directions. For example, to better understand the differences and commonalities between sex differences in both laboratory animals and humans, animal research models have been developed to represent the human condition, such as modeling fundamental diagnostic criteria used in humans (43, 44) and modeling reduced sensitivity to treatment of drug-seeking behaviors, such as escalation (45, 46) and relapse (47, 48). The goal is not only to study sex differences in drug addiction in isolation but also to begin with the foundation of *behavioral dyscontrol* from which it arises, and consider major factors that constitute behavioral dyscontrol, such as impulsive choice and action, compulsive, binge-like drug seeking, and age (adolescence vs. adulthood). Age is especially important because adolescence and young adulthood is when the majority of drug abuse begins. It is ethically difficult to prospectively study this time period in humans; thus, it is essential to work in tandem with laboratory animal models. Biological and behavioral events that occur during this time may be crucial to finding solutions for prevention and treatment of addiction. Another novel approach of this review is to discuss sex differences in *novel treatments* that were developed in animals and tested in humans for drug addiction, and how their effects differ by sex and other vulnerability factors involving impulsive and compulsive behavior. This area has been neglected in most previous reviews, as no viable treatment strategies are currently available to adequately treat human drug addiction. However, the animal literature has begun to reveal several promising leads for prevention and treatment. Section “Sex Differences in the Effect of Novel Treatments for Behavioral Dyscontrol and Drug Addiction” will consider several novel treatments that might be self-sustaining in humans.

Thus, the present review will focus on *sex differences in behavioral dyscontrol*, highlighting key individual differences that can lead to drug addiction. These include *impulsive* drug seeking in the form of impulsive choice and action, such as *compulsive* behavior that leads to binging on drugs when extended access is available, and excessive drug seeking during abstinence that can lead to relapse. The sections that follow will examine sex differences in *impulsive* [for a detailed review, see Weafer and de Wit (49)], and other individual differences such as *compulsive*, binge-like intake of a sweetened liquid (SACC) [see reviews in Ref. (22–26, 28, 31, Carroll et al., under review)], and *age* (adolescence vs. adult) (50). Subsequently, behavioral and pharmacological interventions for reducing these forms of behavioral dyscontrol will be discussed. Table 1 summarizes the vulnerable behaviors that will be discussed as predecessors and predictors of drug abuse, impulsivity, compulsivity, and age (adolescents vs. adults), and how treatment success varies by the vulnerability characteristic. These three vulnerability characteristics will be discussed in Sections “Sex Differences in Impulsivity and Drug Addiction,” “Sex Differences in Compulsive Sweet Consumption as a Predictor of Drug Addiction,” and “Sex and Age (Adolescent vs. Adult) Differences in Behavioral Dyscontrol and Drug Addiction,” respectively, and compared by sex for laboratory animals and humans.

**Table 1:** **Summary of individual differences in selected and selectively-bred rats showing trends in vulnerability to addiction, behavioral dyscontrol, reaction to aversive events, and treatment outcome as a function of sex, age (adolescent vs. adult), impulsivity (I), and sweet intake (S)**.

Vulnerable behaviors	Individual difference	Reaction to aversive events	Treatment outcome	Reference
Drug addiction	F > M	M > F	F > M	Anker and Carroll (6), Becker et al. (17), Carroll et al. (under review), Lynch et al. (51, 263), Perry et al. (53), Anker et al. (54), Cosgrove and Carroll (55)
	Adoles > Adult	Adult > Adoles	Adult > Adoles	Carroll and Anker (7, 263), Anker and Carroll (18), O’Dell et al. (57), Perry et al. (52), Anker and Carroll (56), Spear and Swartzwelder (50)
	Adoles > Adult		Adoles > Adult	Zlebnik et al. (58)
	HMI > LMI		HMI > LMI	Economidou et al. (71)
	HMI > LMI			Diergaarde et al. (77), Belin et al. (78), Dalley et al. (79)
	HiI = LoI		LoI > HiI	Regier et al. (59)
	HiI > LoI			Anker et al. (54), Perry et al. (73), Poulos et al. (75), Diergaarde et al. (77)
	HiI > LoI		LoI > HiI	Broos et al. (69)
	HiS > LoS	LoS > HiS	LoS > HiS	Dess et al. (60, 61), Carroll et al. (25, 28, 62), Perry et al. (29), Anker and Carroll (56), Holtz et al. (63), Holtz and Carroll (23, 24, 64)

Impulsive action	M > F			Jentsch and Taylor (65), Bayless et al. (66), Burton and Fletcher (67)
	HiS > LoS			Anker et al. (68)
	HiI = LoI			Broos et al. (70)

Impulsive choice	F > M			van Haaren et al. (72), Perry et al. (53), Koot et al. (74)
	F = M			Perry et al. (73)
	HMI > LMI		HMI = LMI	Robinson et al. (80)
	HMI > LMI			Broos et al. (70)
	HiS > LoS			Perry et al. (52)

Sweet intake	F > M HiI > LoI			Carroll et al. (25, 28), Carroll and Holtz (22)

*M, male; F, female; HiI, LoI, selected for high vs. low impulsive, delay discounting; HMI, LMI, high and low motor impulsive, 5-CSRTT; HiS, LoS, selectively bred for high vs. low saccharin intake*.

## Sex Differences in Impulsivity and Drug Addiction

Impulsivity, defined as behavior without forethought or consideration of future consequences, is a familiar form of behavioral dyscontrol that has been linked to attention deficit/hyperactivity disorder (ADHD) and is a criterion for substance abuse, pathological gambling, and eating disorders [Ref. (81); see review by Fattore et al. (3)]. Impulsivity is often separated into two main forms: impulsive choice and impulsive action. *Impulsive choice* is defined as a preference for a small-immediate reward over a larger-delayed choice (82), whereas *impulsive action* is considered the inability to withhold a response until an appropriate time is signaled (83). Animal and human preclinical studies have shown that both forms of impulsivity are positively related to drug abuse [for reviews, see Ref. (27, 49, 84, 85)]. There is strong clinical evidence that greater impulsive choice is associated with the development of drug abuse (86), and lower impulsive choice is predictive of better treatment success (87). Results of studies in which both forms of impulsivity have been studied in animal and human research is discussed below.

### Impulsive Choice

Impulsive choice is typically measured using procedures that assess preference for a small-immediate reward over a large-delayed reward over a range of delays to its receipt. One method to quantify impulsive choice is to determine how rapidly an individual discounts the value of the large alternative, as delays are imposed on its receipt. A steeper discounter (more impulsive) would devalue the larger or delayed alternative, and they would shift their preference to the smaller-sooner alternative at shorter delays. A shallow discounter (less impulsive) would tolerate longer delays for a larger reward. Impulsive choice (steeper discounting) is associated with drug abuse and decreased treatment success (85, 88), these findings may partially explain why animal and human females (vs. males) are more predisposed to choose drug abuse vs. healthy alternative behaviors (27, 73, 89).

#### Laboratory Animals

One of the first studies of sex differences in impulsive choice used a Y-maze to assess choice for immediate or delayed food in slightly food restricted rats (72), and females discounted the larger-delayed reward more than males. Subsequently, Perry et al. (52) conducted a similar study of impulsive choice for food using a two-lever operant conditioning chamber in which responding on one lever resulted in a small-immediate amount of food and responding on the other lever produced a larger-delayed amount. When this experiment was replicated with other groups of male and female rats that self-administered i.v. cocaine under a similar delay-discounting task, there were no sex differences. This was likely due to a ceiling effect since overall impulsivity for cocaine was much higher than for food (52). In a subsequent study, Perry et al. (73) compared male and female rats selected for high vs. low impulsivity (HiI vs. LoI), based on the delay-discounting task for food, on *acquisition* of cocaine self-administration, and on cocaine-primed reinstatement of cocaine seeking (a model of relapse). They found that both HiI males and females acquired cocaine self-administration faster than LoI males and females, and HiI females showed greater cocaine seeking during reinstatement than LoI females or either group of males (Hi, LoI). A similar study was conducted by Koot et al. (74) in mice that were divided into steep (more impulsive) vs. shallow (less impulsive) discounters based on a median split. Within the steep-discounter group females were more impulsive than males. Overall, while there are only a few studies of impulsive choice in animals, the results consistently support a moderately higher level of impulsivity in females than males.

#### Humans

In humans, sex differences in impulsive choice are more mixed. Women tend to be slightly more impulsive than men when making choices between real (lottery based) and hypothetical monetary outcomes [Ref. (90–93); but see Ref. (94)]. However, in some studies, no differences have been reported (95–100). Kirby and Maraković (94) reported that men were more impulsive than women when hypothetical money was a choice, but when real money was based on a lottery, men discounted money more steeply than women at the higher monetary values. Overall, women generally show greater impulsive choices for hypothetical rewards, but men show more impulsive choices for actual rewards.

In summary, regarding impulsive choice, results of animal studies suggest that females exhibited more impulsivity than males in the transition states of addiction, such as initiation and relapse or reinstatement of drug-seeking behavior. In humans, the methods are quite different than in animals, but women were more impulsive toward hypothetical rewards, and men were more impulsive for actual rewards [see Ref. (49)].

### Impulsive Action

*Impulsive action* is typically considered an inability to inhibit non-productive or inappropriate responses (83), and in humans and animals, two tasks to quantify this form of impulsivity are commonly used, the stop-signal reaction time (SSRT) and Go/No-go tasks. These tasks signal periods of responding and non-responding, and failures to inhibit an inappropriate response are considered instances of impulsive action. During the SSRT, a subject must inhibit an ongoing “go” response when a “stop” signal is presented, making the task more difficult than the Go/No-go task in which the participant must respond to a “go” stimulus but inhibit a response following “no-go” stimulus. In these tasks, researchers consider *impulsive action* to be an increase in errors of commission (i.e., failures to inhibit responding to an inappropriate stimulus in SSRT, more responding during a no-go period, and longer stop-signal reaction times). These tasks are similar to the relapse aspect of drug addiction, whereby individuals are unable to withhold responding to drug-related cues (e.g., accepting a drink offer).

#### Laboratory Animals

In an animal study of impulsive action, male and female rats were compared on a Go/No-go task for food or i.v. cocaine infusions (68), and no differences were found in responding for food reward during the no-go period (impulsivity measure). However, females made more responses for cocaine infusions during the no-go period than males, and this was consistent with measures of impulsive choice (52). In the five-choice serial reaction time task (5-CSRTT), no sex differences were found in mice during acquisition or the challenging portion when long intertrial intervals (ITI), stress, and *ad libitum* food were tested (98). However, over repeated testing, females were more impulsive than males as indicated by premature responding (action impulsivity). In another 5-CSRTT study of young vs. adult rats, no sex or age differences were found in task acquisition, but females made more premature responses than males in the challenging task (long ITI) (67).

As in a previous study of impulsive choice (51), sex hormones were implicated in studies of impulsive action. For example, Jentsch and Taylor (65) compared intact and gonadectomized, male and female rats, in a 5-CSRTT study, and intact males made more premature responses than gonadectomized females during the acquisition and challenge (long ITI) conditions. Gonadectomy increased impulsive action in males and ovariectomy increased impulsive action in females, suggesting that both testosterone and estrogen are related to impulsive action in rats. In a recent study by Bayless et al. (66), comparing male and proestrus female rats on the 5-CSRTT, males showed greater impulsivity (premature responding) than females. Thus, there is an indication that sex and hormonal status are factors in measures of impulsive action.

#### Humans

In tests of impulsive action with humans, sex differences have been mixed, depending on the procedure employed. Under the Go/no-go procedure, males tended to commit more inhibitory errors than females (99, 100), but in other studies, there were no sex differences (93, 101). Similar findings occurred using a continuous performance task (CPT). In an eight-study meta-analysis of children with ADHD, boys consistently made more errors of commission (i.e., more impulsive) than girls (102), and there were similar findings in adolescent vs. adult smokers (103). However, in the SSRT, females had longer reaction times (more impulsive) than males (103–106); although, in a similar number of studies, no sex differences were found (93, 101, 106, 107). Thus, human sex differences in impulsive action may be procedure dependent. Males were more impulsive on tasks requiring inhibition of ongoing “go” responses (e.g., CPT and Go/No-go task), and females were more impulsive on the SSRT task (i.e., longer reaction times) that requires initiation of a response.

Sex differences in impulsive action also extend to drug addiction, and women drug users are more impulsive than men. Female heavy drinkers and adolescent smokers were more impulsive than males on the SSRT (103, 106) and CPT (108) tasks. Interestingly, the non-drug-seeking control males were similar or more impulsive than the female controls, suggesting a strong covariance of impulsive action and drug abuse in females vs. males. Estrogen reduced impulsive behavior in a SSRT task in humans (104), and women in the follicular phase, when estrogen levels are peaking, were more impulsive (longer reaction times) than during the luteal phase when the estrogen levels are low and PRO levels peak and decline. Overall, sex and sex hormones play a role in modulating impulsive action. Specifically, PRO reduced impulsivity (Smethells et al., under review); thus, using PRO as a treatment to target impulsivity may be effective for reducing addictive behavior (see Targeting Individual Differences with Repurposed Medications as Treatments for Addiction).

A general trend in sex differences in impulsive action in animals and humans is less clear than for impulsive choice; however, a wider range of tasks are used to assess impulsive action, and they may be accessing different elements of the behavior. In animals, males exhibit more impulsive action than females, but it is task dependent. In humans, women tend to show more impulsive action than men on several tasks [see Ref. (49)].

## Sex Differences in Compulsive Sweet Consumption as a Predictor of Drug Addiction

For many reasons (e.g., television advertising, changes in food production, and fast food access), changes in the U.S. culture over the last century have led to a condition where two-thirds of the population is overweight or obese, resulting in premature death due to metabolic syndrome, diabetes, heat disease, digestive disorders and associated cancers (109). The terms “food addiction,” “hedonic overeating,” and “food insecurity” (110) have been used recently to describe and explain chronic overeating leading to weight gain, and these concepts highlight underlying similarities between excessive eating and drug addiction. However, there is some disagreement that the behavioral and neurobiological mechanisms underlying this behavior are completely parallel to those involved with drug addiction [e.g., Ref. (3, 11, 12, 22, 28, 111–113)]. Nevertheless, recognizing similarities between “drug” and “food” addiction may be useful for designing treatment strategies.

Animal models have also been useful in understanding behavioral dyscontrol in food consumption as it relates to drug addiction. For example, sugar-binging rats extended this behavior to amphetamine (114), and rats selectively bred to consume large amounts of a SACC-sweetened liquid (HiS) showed faster initiation of heroin self-administration (62), with more animals per group acquiring drug use, escalating, and relapsing after forced abstinence, than rats bred for low SACC intake (LoS) (28). Other studies that model criteria for addiction in humans indicate that the HiS (vs. LoS) rats meet several DSM V (81) criteria for addiction, such as tolerance, difficulty limiting use, spending excessive time-seeking drug (29, 30), escalation of drug intake (115), impaired ability to regulate drug intake (51, 116), and continued use despite aversive consequences (117). HiS rats also showed impaired ability to regulate SACC intake (51). While sweet preference predicts all aspects of drug addiction, only a few studies in animals and humans have reported sex differences in these behaviors (28).

### Laboratory Animals

One of the best examples of animal models of behavioral dysregulation in feeding that is related to drug addiction and obesity is from studies initiated by Dess and colleagues. They bred different lines of rats that ingested excessively high levels of a sweet SACC solution (HiS) or low to normal amounts of SACC (LoS). Their early studies also revealed greater sensitivity in HiS vs. LoS rats to several tastes, such as sweet, salty, and bitter (118), and they found more ethanol intake in the HiS than the LoS rats (61, 119). Subsequent studies with the HiS/LoS rat lines in our laboratory with cocaine or heroin showed that HiS rats exceeded LoS rats during all phases of drug addiction, including initiation, escalation, or binging on cocaine during long access, resistance to extinction when cocaine availability was terminated, and relapse or reinstatement of drug seeking instigated by brief exposure to cocaine, stress, or cocaine-related cues, even several weeks after cocaine self-administration had terminated [see reviews in Ref. (22, 25, 26, 28)]. In a recent study in our laboratory, the findings of SACC preference predicting drug self-administration were extended to other measures of reward, such ICSS, and HiS rats showed more cocaine-induced reward enhancement of intracranial self-stimulation than LoS rats (120). Converging results from many of these studies suggest that avidity for sweets and drug-taking behaviors are closely related, heritable, and substitutable (22, 25, 114), and they likely operate through common neural mechanisms [e.g., Ref. (121)].

A similar connection between sweet preference and drug addiction (e.g., HiS vs. LoS) was also found with outbred rats that were selected for high or low intake of SACC [e.g., Ref. (122, 123)], or other sweet substances, such as sucrose (124, 125), and opioid self-administration (126). The results were similar to those obtained in the HiS vs. LoS rat studies. The connection between drug addiction and overindulgence in food was recently modeled in a study by Yakovenko et al. (127) in which HiS rats exhibited more binge-like behaviors with access to high-fat or -sugar containing substances than LoS rats. This strong predictor of drug abuse has also been found to interact with sex. Sex differences in drug-seeking behavior were also examined in the HiS vs. LoS rats, and females exceeded males on drug seeking and intake. During the acquisition phase of drug self-administration, HiS and LoS females exceeded males in ethanol intake (119). During maintenance, females also consumed more ethanol (61, 119) and heroin (62) than males. HiS females also scored higher than LoS females on cocaine-induced locomotor activity and cocaine-induced sensitization of locomotor activity (116). Thus, HiS and female rats showed more cocaine-induced locomotor activity and sensitization than LoS and male rats.

Overall, the comparisons of HiS vs. LoS rats indicated that sex and SACC preference were additive predictors of behavioral dyscontrol in the form of drug addiction. Across several studies, the HiS females were ranked highest in drug seeking, followed by LoS females or HiS males, and then LoS males were lowest in terms of drug intake (22–25, 28). When the results of these studies were translated into DSM V (81) criteria for addiction (43), HiS rats exceeded LoS rats on several addiction criteria, such as tolerance, difficulty limiting use, excessive time-seeking drugs, impaired control over use, and despite punishment, as well as resistance to withdrawal effects [see reviews in Ref. (22–26, 28)]. Thus, the animal findings lend strong support to the conclusion that drug and food addiction have many similar characteristics.

### Humans

In humans, parallels between food and drug addiction are beginning to emerge, but these areas have remained separate in the feeding literature, except for a few isolated reports. The relationship between substance abuse disorders and avidity for sweets has also been reported in human alcohol (128), cocaine (129), and opioid abusers (130). However, a more recent trend in hedonic overeating, often called “food addiction” that results in overweight and obese individuals in nearly two-thirds of the U.S. population, is nearly equally distributed in males and females (131) or worldwide, slightly more prevalent in women than in men (13). Other eating disorders, such as bulimia and anorexia nervosa, have not been closely studied for sex differences, as most eating disorders have predominantly occurred in females [see edited volume by Avena (132)], but the lack of sex differences in the Centers for Disease Control and Prevention (131) data may be due to several factors, such as food is an essential commodity, whereas addictive drugs are optional. Nevertheless, the parallels and differences between addiction to food vs. drugs may be informative for prevention and treatment strategies. Currently, there is little human literature regarding an interchange between food and drug addiction, and how that might vary by sex. However, this interchange has been clearly demonstrated in rats, and females exceed males on both hedonic overeating and drug addiction (22, 28). The translational implications for humans are that as weight loss is effectively pursued, individuals will be at higher risk for drug addiction, and this is more likely to occur in females than males.

In summary, while animal data clearly indicate a strong connection between food- and drug-seeking behavior, and females show more drug seeking and sweet preference than males, there are not enough data available at this time to determine whether parallels in hedonic overeating and drug addiction extend to humans.

## Sex and Age (Adolescent vs. Adult) Differences in Behavioral Dyscontrol and Drug Addiction

Age (adolescence vs. adult) is an important individual factor to consider when evaluating the contribution of sex and behavioral dyscontrol to addiction, because adolescence is when biological (hormonal) and behavioral (impulsivity, risk-taking) changes emerge in animals and humans, and these are major variables contributing to drug addiction. Laboratory animal and human adolescents and adults have been compared and reviewed in several previous studies of drug addiction for their differential responding to both the rewarding and aversive aspects (136–133). Generally, adolescents are more sensitive to the rewarding effects of drugs of abuse, but they have reduced sensitivity to the aversive effects. Importantly, animal and human studies indicate that adolescents are also more sensitive than adults to other major factors included in this review that have been noted to predict drug addiction, such as impulsivity (56, 134) and compulsive sweet intake (134–136). Sex differences in the development of addictive behavior are difficult to study during the adolescent period in animals, as adolescence is only about 30 days in rodents. In humans, there is mostly epidemiological research on behavioral dyscontrol and adolescence vs. adulthood, which has been informative, but prospective studies are limited due to the difficulty of studying human adolescents. The following sections review age-dependent effects of alcohol use in animals and humans, since it is a widely abused and well studied in the adolescent population (2).

### Laboratory Animals

The animal literature indicates that adolescent rats self-administer about two to three times more alcohol than adult rats [e.g., Ref. (137–139)]. Research with rats has also established that early alcohol consumption in adolescent-exposed rats produced more impulsive risky choices in adults, compared to control rats that did not have adolescent alcohol access (140, 141). Contrary to human studies, female rats tend to consume more alcohol during adulthood than adolescence [e.g., Ref. (138, 142)]. The higher intake of alcohol in male adolescent rats, compared with adults, is likely due to a reduced sensitivity to the alcohol-induced sedative/hypnotic (143), hypothermic (144, 145), motor impairing (146), anxiolytic (147), and anxiogenic effects (148). This decreased sensitivity combined with a slightly higher rate of alcohol metabolism potentially enables adolescent rats (and perhaps adolescent humans) to consume more alcohol (144, 145, 149). While adolescent rats were more vulnerable than adults to various forms of addiction (150–152), opposite age effects have been reported (133, 153). Several studies have investigated the effect of adolescent drug exposure on subsequent adult drug use in animals (56) and humans (154, 155), and the findings indicate that early exposure facilitates adult drug abuse. Few studies have compared sex differences in rat studies of adolescence and addiction. In one study, rats self-administering cocaine were exposed to physical exercise as a treatment, and it was more effective in adolescents than adults (58). More details on treatment are presented in Section “Physical Exercise.”

### Humans

Although few studies have compared sex and age with respect to drug addiction, one study indicated that in humans, young-adults (ages 18–25) drink more alcohol than older adults (ages 35–54) [e.g., Ref. (156)]. For instance, in the Naimi study, binge drinking (>5 drinks per sitting) occurred about two to three times more frequently in younger adults than older adults, with males far exceeding females across all age groups. Age of exposure to alcohol also interacted with other risk factors for drug use, including impulsive and risky behavior. It has been found that those whose initial alcohol problems began in adolescence (age 13–17) are more impulsive than controls. Impulsivity was observed in early drug use (157–160) and later (38–46 year olds) after drug use had become fully established (161–166). These results suggest that exposure to alcohol early in life may increase impulsive and risky behavior, and adolescence may be a critical period when drug use alters prefrontal brain development leading to increased impulsivity [Ref. (167); see reviews by Brown and Tapert (168)]. The earlier the age of initial alcohol exposure, the poorer the prognosis for alcohol abuse in adulthood, and this can result from ease of access. For example, in the case of nicotine, second-hand smoke in children and adolescents yields nicotine content similar to actual smoking (169). Thus, parental smoking can accelerate health risks from smoking in addition to smoke inhaled by adolescents who use tobacco.

Dom et al. (164) found that the age of an alcohol problem onset was important for increasing impulsive choice that is predictive of further drug use. The rate of this increase when compared to controls, however, was only significantly steeper (i.e., impulsive choice was greater) for alcoholics whose alcohol problems started earlier in life (<25 years old) but not for alcoholics whose alcohol problems started later in life (>25 years old). Given the small number of females included, sex differences could not be determined; however, the findings suggested an age-dependent relationship between the onset of an alcohol problem onset and impulsive choice. Thus, early alcohol exposure may cause increased levels of impulsive and risky behavior later in life leading to more drug abuse.

Taken together, the animal and human research suggests that age (adolescence vs. adults) is a significant vulnerability factor that interacts with other major factors, sex and impulsivity, and since males consume more alcohol than females during adolescence, this may result in enhanced vulnerability for alcohol abuse in males later in life.

## Sex Differences in the Effect of Novel Treatments for Behavioral Dyscontrol and Drug Addiction

In previous attempts to develop treatment for drug addiction, receptor pharmacology guided medication development. Several medications were designed to act on transmitter systems involved in drug addiction, and a consistent finding in these studies was that the treatments were more successful for female than male animals. For example, female rodents showed a greater reduction in cocaine self-administration than males when treated with kappa opioid agonists, spiralodine (170), bremazocine (171), a GABA_B_ agonist, baclofen (172), a corticosterone synthesis inhibitor, and ketoconazole (173). In more recent studies with rats modafinil (an analeptic drug) decreased methamphetamine (METH) induced reinstatement (relapse) in both males and females. Other studies compared treatment effects bremazocine, a kappa opioid receptor agonist, in female and male monkeys self-administering orally delivered PCP (55), while females consumed more drug than males (milligram per kilogram), they reduced their drug intake more than males with bremazocine (171) treatment (see Table 1). However, most of those treatments failed to show efficacy or had undesirable side effects when translated to humans.

Despite these previous innovative treatment attempts and their success in animals, there are currently no safe, non-addictive, effective treatments for reducing the morbidity and mortality of drug addiction that are useful in humans, except for agonist therapies (e.g., methadone, buprenorphine) and drugs that have modest effects on relapse to smoking (e.g., varenicline – Chantix) or alcohol abuse (e.g., naltrexone). This is indicated by epidemiological reports that the rates of most forms of addictive behavior have remained steady or increased over the last decade, and there are endless new forms of addiction (e.g., designer drugs, bath salts, etc.) that defy treatment (32). Thus, development of treatments for drug addiction is a high priority. Of the studies that show some promising initial findings, very few have compared males and females. A review of 280 treatment studies for substance abuse disorders in men and women that were published between 1975 and 2005 indicated better treatment outcomes for women than men (174). However, their later analysis of the multi-site combined pharmacotherapy and behavioral interventions for alcohol dependence program (COMBINE), including 1383 men and women, reported that while there were sex differences in those seeking treatment for alcoholism, there were no sex differences in the combined treatment condition. Women responded to naltrexone treatment combined with a medical management control condition similar to men (175).

A novel approach to designing new treatment strategies is to target factors that underly drug addiction. For example, behavioral dyscontrol is common to many forms of addiction; thus, treatment models can be designed to remedy this underlying aspect of drug abuse. The reinstatement (relapse) model has been useful for this purpose, as it portrays several aspects of the drug addiction process that occurs in humans, such as *acquisition* or initiation of drug self-administration, steady *maintenance* intake, *escalation or binge-like* intake of drugs, persistence of drug seeking (drug-lever responding) during *extinction* or *abstinence* when the drug is no longer available (*compulsive drug seeking)*, *reinstatement* or *relapse* of drug seeking following experimenter-administered injections of the drug or presentation of drug-related cues or stress stimuli, and *incubation of craving* (a time-dependent increase in drug seeking) that accelerates drug craving and leads to relapse after extended periods of abstinence (176, 177). Earlier studies with rats and rhesus monkeys indicated that behavioral interventions as well as medications have had some success in reducing drug-motivated behavior, and some of these studies indicated that females were more responsive to treatment than males [see review by Carroll and Holtz (22)].

Much of the animal findings regarding medications for drug addiction have generally not translated to effective treatments for drug abuse in humans. Thus, recent animal studies have focused on novel treatments for drug addiction that could be self-sustaining in humans. These include (1) *using natural consequences* such as *non-drug rewards* or positive events (environmental enrichment) that a drug-abusing individual might encounter in the environment that would compete with drug use (e.g., social interaction, exercise). Also, negative consequences, such as punishment for drug use are naturally built into the environment and can be programed to reduce drug use. (2) *Targeting factors that underlie behavioral dyscontrol*, such as *impulsivity or anxiety* by repurposing medications designed to relieve these underlying behaviors that can drive drug addiction. For example, PRO [e.g., Ref. (40)] or atomoxetine (ATO) could be used for anxiety, impulsivity, or other forms of behavioral dyscontrol that are associated with ADHD, and (3) combining two or more novel behavioral and pharmacological treatments.

### Environmental Enrichment

A widely studied and promising approach for reducing or preventing the development drug addiction (as a form of behavioral dyscontrol) has been to enrich the environment with non-drug rewards [see reviews in Ref. (27, 28, 178, 179)]. This has been a successful treatment method for reducing many aspects of drug addiction, and it is well supported by extensive preclinical and clinical evidence. However, this method has not been widely studied with respect to individual differences, such as sex. In earlier studies, a commonly used method of environmental enrichment for reducing drug-seeking behavior was to use preferred foods (180), or place animals after weaning in a larger social environment (vs. isolated) that contains novel objects and activities (181). Non-caloric sweet substances (e.g., SACC) were also effective as competing rewards to reduce drug seeking in rats [e.g., Ref. (55)] and rhesus monkeys [e.g., Ref. (182, Carroll et al., under review)]. In these environmental enrichment studies, females reduced drug taking more than males when they had sweet substances concurrently available [see reviews in Ref. (22, 25)], or when they had prior access to a sweet substance (183, 184). Studies with female and male monkeys self-administering orally delivered PCP (55) or cocaine (Carroll et al., under review) indicated that females consumed more drug than males (milligram per kilogram), but females also reduced their drug intake more than males when treated with access to a non-drug reward, SACC (see Table 1). While these therapeutic advances were effective and providing palatable substances was a powerful intervention for drug abuse [see Ref. (22–25, 28)], more recent studies have sought to provide a healthier environmental enrichment alternative, focusing on social and physical elements of the environment.

#### Social

Taking drugs in a social environment is important for humans and non-human primates. In behavioral economic terms, some drugs and social rewards work together as complements, and each increases the other, such as drinking and smoking at a social gathering, or smoking while talking on the phone. However, in other cases, social stimuli and drug-taking work as substitutes, whereby one reward may replace the other (173, 185). Thus, the rewards of social interaction can be used as substitutes to reduce drug taking (181).

##### Laboratory Animals

Rearing environment is an important factor in the development of drug self-administration. To examine this, rats were raised in enriched conditions (EC) with a large environment, several cage mates, and a variety of toys and exercise devices, whereas rats raised in the isolated condition (IC) were singley housed in smaller standard rat cages. As adults, rats were allowed to self-administer drugs, and EC rats self-administered less amphetamine than the IC rats (181). Lower rates of responding in EC rats (vs. IC) indicated that the enriched environment reduced motivation for amphetamine (lower break point on a progressive ratio schedule) (186). The EC rats were also less impulsive during the acquisition of an impulsive action task compared to IC rats (187), and they were less impulsive than IC rats on an impulsive choice procedure (73). These findings suggest that early exposure to an enriched environment may alter sensitivity to drugs of abuse and blunt the development of drug abuse in adulthood; however, sex differences were not often considered in these studies.

##### Humans

In humans, non-drug rewards delivered in a contingency management (CM) format successfully reduced drug dependence [for a review see Ref. (188)]. In general, CM programs promote drug abstinence through a combination of positive reinforcement for drug-free urine samples. For instance, voucher-based reinforcement therapy in which medication compliance, therapy session attendance, and negative drug screenings reinforced with vouchers to local business (e.g., movie theater, restaurants, etc.) directly reinforces drug abstinence, provides competing reinforcers, enriches the environment, and it is a robust treatment across a broad range of abused drugs (189). Another example of using social rewards to reduce drug addiction was given in the Naimi et al. (156) study, comparing younger and older adults, who reported that enhancing non-alcohol-related campus social programing had decreased alcohol use.

In summary, both animal and human studies indicate that environmental enrichment is an important intervention that moderates the development and progression of drug addiction. There is little information regarding sex differences in social reward at present; however, once drug use patterns have developed, non-drug rewards, such as social interaction, have the advantage of being self-sustaining and are effective in both sexes.

#### Physical Exercise

There is accelerating evidence that physical exercise is a useful treatment for preventing and reducing drug addiction [see reviews in Ref. (28, 178, 190, 191)]. In some individuals, exercise has its own rewarding effects, and a behavioral economic interaction may occur, such that physical and social rewards of exercise can substitute for the rewarding effects of drug abuse. Exercise has also been a valuable treatment for slowing cognitive decline in patients with dementia [e.g., Ref. (192)], health-related problems in obesity [e.g., Ref. (193)], and in psychiatric disorders, such as anxiety (194), depression (195), and schizophrenia (196). The value of this form of treatment for drug addiction in laboratory animals and humans is that exercise, if it can substitute for the rewarding effects of drugs, could be self-maintained over an extended period of time. Work to date in laboratory animals [for review, see Ref. (191)] and humans [for review, see Ref. (178)] regarding exercise as a treatment for drug addiction supports this hypothesis.

##### Laboratory Animals

Recent animal studies have consistently reported that exercise reduces drug-seeking behavior in both self-administration and conditioned place preference (CPP) studies [see reviews in Ref. (28, 178, 190, 191)]. In rat studies, exercise in the form of wheel running decreased cocaine-seeking behavior in males and females across all phases of the drug addiction, including acquisition (197), maintenance (58, 198–201), escalation/binging (58, 201, 202), extinction (203–205), and reinstatement/relapse (203–207), including extended relapse or incubation of cocaine-cue-induced reinstatement (craving) over extended time periods (208). Voluntary running is also effective if it is provided in the home cage environment, and drug-seeking behavior is tested separately in an operant chamber [e.g., Ref. (203, 204, 206, 208)].

There have been few studies directly comparing sex differences on the effects of exercise as a treatment to reduce drug-seeking behavior [see review by Zhou et al. (190)]; however, limited evidence shows that concurrent access to a running wheel (vs. a locked wheel) reduced cocaine self-administration more in female than male rats (198). Few studies have compared sex and age in treatment studies with rats. However, in rats self-administering cocaine, physical exercise was more effective in adolescents than adults (58). Exercise may be a more suitable treatment than pharmacological interventions in adolescents who are undergoing critical phases of development and brain maturation (209, 210). In animal studies, both concurrent exposure to exercise (198, 211) and prior exposure and/or exposure in a different environment (200, 201, 203, 204, 212–215) effectively reduced drug seeking.

While both *concurrent* and *sequential* approaches are effective, these data show actual reductions in drug intake (vs. drug seeking) with *concurrent* access to exercise (198, 211) and other non-drug rewards (28), while previous studies using *sequential* access to exercise report that initiation of drug self-administration (213) or drug-seeking behavior during extinction from former access (relapse) is suppressed [e.g., Ref. (212)]. While concurrent and sequential access to drug and exercise has not been directly compared in rat studies, there may be an advantage to allowing concurrent access or at least presenting both in a contiguous time frame. For example, a previous within-subjects study using treatment with a non-drug reward (SACC) in monkeys, with both concurrent and sequential access, verified a more robust reduction in drug intake with *concurrent* access to SACC than sequential access (183). Thus, comparing concurrent vs. sequential access, and contingent access [e.g., Ref. (188)] with exercise as a treatment is an important area for future research.

Recent studies in rats have examined sex differences on the effect of previous exercise exposure in a different environment on subsequent drug seeking during different parts of the drug addiction process. Ehringer et al. (199) indicated that females significantly lowered their alcohol consumption compared to males when a running wheel was available, but not during the reinstatement (relapse) component. Smith et al. (204) did not find a sex difference in the effect of wheel running on cocaine self-administration or reinstatement, but they found that females decreased drug seeking more than the males during the first few extinction sessions when a running wheel was available. However, two studies directly compared the effect of exercise in male and female rats self-administering cocaine (198) or on cocaine-primed reinstatement (216), and both found a better effect of exercise in reducing drug seeking in females than males. In other studies, wheel running reduced cocaine (206) and nicotine acquisition (213) and nicotine seeking during reinstatement (212). Nevertheless, in the cocaine study (206), males’ cocaine seeking was also reduced more than females’ by entry into the locked wheel control condition, and an opposite sex difference was found in the nicotine study (212) whereby females’ nicotine seeking was reduced more than males by entry into the locked wheel control condition. In contrast, Smith et al. (204) did not find a sex difference in the effect of wheel running on cocaine self-administration or reinstatement, but they found that females had decreased extinction responding compared to males. Results of these and other initial studies [e.g., Ref. (58, 198, 205, 216, 217)] suggest that the effects of exercise are strongest when exercise is available during the critical phases of addiction (acquisition, maintenance, escalation, or drug-primed reinstatement), and sex differences (F > M) are found. More work is needed with both males and females during all phases of addiction to identify the most effective treatment strategy. While numerous studies exercise as a treatment for addiction have been conducted with both male and female rodents [see Ref. (191); Table 1] using both drug self-administration and CPP models, approximately 80% of the work has been done with males. It was encouraging that in most of the studies reviewed, exercise had an advantageous effect on preventing or treating CPP for the environment where drug exposure occurred.

In general, existing studies suggest that physical exercise is an effective deterrent to drug seeking and abuse, and it offers a healthy, self-sustaining treatment for drug abuse. However, more work is needed to evaluate the potential for this treatment in both males and females and its effect on individuals with other vulnerabilities for drug abuse. Moderate use of this treatment may be the key to its success. For example, non-drug rewards such as excessive amounts of sweet drinks also reduce drug addiction in animal models (185), but they can also become addictive (112, 218) and lead to other unhealthy consequences. Similarly, while it is uncommon, too much exercise could result in health issues, such as exercise addiction and exercise-induced anorexia (3, 219).

In summary, emerging evidence from the animal literature indicates that exercise is a healthy candidate for treating drug abuse, but not enough data are available to make a strong prediction regarding sex differences in treatment efficacy or the best strategy for delivering this treatment, whether it is concurrent with drug access, sequential, or contingent upon non-use of drug [e.g., Ref. (188)]. In previous rat, monkey, and human studies, concurrent and/or contingent access to drug and non-drug rewards have been the most effective strategies for reducing drug abuse [see Ref. (173, 178, 185, 188)].

##### Humans

Compared to the large number of laboratory animal studies that have prospectively examined physical exercise as a potential treatment for drug abuse [see Ref. (191)], human studies are few, and the results are not as definitive. Most of the human data are cross-sectional, but importantly they involve cigarette smoking, which is easier to study than illicit drugs because large sample sizes are available and it is a legal drug. However, in a recent review of the clinical literature, Linke and Ussher (220) concluded that there is a lack of prospective randomized clinical trials (RCT) that are needed to study the effects of exercise not only nicotine, tobacco, and alcohol abuse but also for other drugs that have a high rate of abuse, such as METH. For example, in several studies, higher abstinence rates were reported at 3 months (195, 221, 222), 6 months (223), and 12 months (221) after an exercise regimen; however, other studies found no significant effects of exercise on abstinence (220, 224). In a recent review of the literature on physical activity and drug abuse, Bardo and Compton (178) noted that the impact of physical activity on the reduction of drug intake in humans has also been shown mainly in observational studies, both cross-sectional and prospective. Survey research has also indicated that higher levels of physical activity are associated with lower alcohol, tobacco, and marijuana use (225).

Reviews of these correlational studies emphasize a need for RCT in alcohol, tobacco, and marijuana addiction, and initial studies on the use of exercise programs for treatment tobacco use have shown improvement for smoking cessation [e.g., Ref. (223)]. However, others have shown no benefit, possibly because they were underpowered. There are efforts to promote physical activity as an adjunct for smoking cessation, especially among women (224), but key parameters, such as type and intensity (dose) of physical activity, have not been determined. Aside from the few studies on tobacco and alcohol, there are no reports of RTC studies showing improvements in outcomes on drug addiction using exercise as an inpatient treatment. However, a RTC study was recently reported by Rawson et al. (226), whereby they used 8 weeks of exercise as a post-residential treatment for METH addiction, showed a significant reduction in use (confirmed by urine screens) in participants who had been using meth 18 days or less a month. Earlier reports from this group showed that exercise also resulted in improvements in fitness and heart rate measures (227, 228). In another human study on cigarette smoking, it was reported that individuals were more successful in maintaining abstinence if they continued their exercise program on their own after the experimental intervention ended (221).

Animal and human research on physical exercise as a treatment for stimulant addiction indicates that this is one of the most promising treatments on the horizon. However, there are few studies of sex differences in outcome of this form of treatment. Initial animal work suggests that females and adolescents are more responsive to this form of treatment than males; however, further animal work and extension to human RTCs is needed.

### Negative Environmental Consequences

For drug abusers, punishment exists in natural settings, in the form of natural consequences for drug use, such as loss of friends, jobs, money, and to promote survival. It has seldom been proposed for treating drug abuse in humans, although treatment methods for alcoholism, such as antabuse, re-setting voucher amounts in VBRT after positive urine samples, and revocation professional licenses for drug addition, are forms of punishment that human drug abusers encounter. While treatments based on negative environmental consequences have not been systematically explored in humans, animal studies indicate that negative consequences for drug use may be an important aspect of treatment to consider. However, only a few animal studies have modeled the effect of punishment on drug seeking and drug self-administration, and results indicate that mild forms of punishment are effective and enduring. For example, after several months of ethanol intake, rats continued to drink alcohol despite the consequences of footshock (229) or bitter tasting quinine (230), and this aversion-resistant alcohol intake is considered to be a model of compulsive drug abuse in humans [e.g., Ref. (231)]. However, in some animals, these aversive pairings with drug self-administration reduce drug intake. The extent to which rats have reduced sensitivity to aversive effects of drugs interacts with individual differences, such as sex, age (adolescent vs. adult), sweet preference (HiS, LoS), and impulsivity (HiI, LoI). Given the individual differences in vulnerability to addiction (see Table 1), and response to treatment effects in rats and monkeys with biologically and behaviorally mediated differences (male/female, HiI/LoI, HiS/LoS and adolescent/adult), recent animal studies have considered individual differences in response to punishment as a treatment for drug abuse. Histamine was used as a chronic, aversive condition to validate a model of punished drug seeking that would represent the negative emotional and physical symptoms (hangovers, anxiety, anhedonia, and irritability) experienced by humans. Histamine (i.v.) was added to the i.v. cocaine self-administration in groups of male vs. female HiS vs. LoS, HiI vs. LoI, and adolescent vs. adult rats (23, 117). All groups suppressed responding for cocaine when histamine was added. Female and LoS rats showed a significantly slower (5–15 days) return to baseline levels of cocaine self-administration after histamine was terminated, and HiI and LoI rats showed no differences throughout the experimental phases (117). However, while adult rats also showed a greater punishment effect than adolescent rats when histamine was present in the cocaine solution, adults and adolescents recovered to baseline at the same rate (23).

Consistent with the histamine findings, in other studies, adult rats had more severe withdrawal effects than adolescent rats (232, 233). This was in contrast to findings that adolescent rats self-administering cocaine were more sensitive to the rewarding effects of drug (52, 120) and showed more severe relapse effects than adult rats (18). These findings highlight opposite effects that can occur in groups of rats when considering the rewarding vs. aversive effects as previously discussed by Riley (234), and they emphasize the importance of considering individual differences in vulnerability to drug abuse and response to treatment. These results with differentially vulnerable groups concur with recent treatment studies with baclofen, an agent that reduces cocaine-induced dopamine increase in the nucleus accumbens. Baclofen treatment reduced cocaine self-administration in the less vulnerable LoS animals, and potentiated it in the more vulnerable HiS animals (63). Similar effects were found with PRO that reduced escalation of cocaine self-administration in LoS rats and increased it in HiS rats (56). These studies highlight the importance of considering novel treatment mechanisms and individual differences in response to different treatments.

### Targeting Individual Differences with Repurposed Medications as Treatments for Addiction

In recent studies, proposed novel treatments have addressed factors that underlie behavioral dyscontrol. For example, (1) impulsivity has been shown to be positively related to drug addiction, and repurposing medications that reduce impulsivity to treat underlying problems had initial success in treating drug addiction, as both male and female humans report that it reduces anxiety. For instance, ATO that is used to treat ADHD, and it reduced impulsivity in rats (235). (2) Hormonal conditions are known to increase (estrogen) or decrease (PRO) cocaine and nicotine-seeking behavior, especially in females, and PRO has emerged in animal and human studies as a promising medication that could be repurposed for drug addiction, as both male and females report that it reduces anxiety. For example, PRO is used in some oral contraceptives to treat problems with the female reproductive system, but when used for drug-abuse treatment, it counteracts the facilitatory effects of estrogen and reduces drug relapse [see Ref. (9)]. PRO also has anxiolytic effects that reduce drug seeking [e.g., Ref. (40, 236)]. (3) An additional strategy has been to combine two or more novel approaches, such as medication (e.g., ATO, PRO) or behavioral treatments, that often has a greater impact than monotherapy in animals and humans.

In summary, research in animals has begun to target specific behaviors or hormonal conditions that are associated with addictive behavior, such as anxiety, depression, and impulsivity. In this section, we discuss two repurposed medications, ATO and PRO, as they have shown efficacy for treatment in rodent studies. Thus far, the results support the hypothesis that treating the underlying behaviors associated with drug abuse, with PRO and ATO, has potential for treating human drug abuse, and as discussed in Section “Treatment Combinations,” adding these treatments (ATO or PRO) to a behavioral treatment in rats, such as physical exercise, results in an enhanced treatment effect. However, initial studies with these novel treatments have not fully examined sex differences, and sex is an important factor in drug abuse and its treatment.

#### Atomoxetine

Atomoxetine is a selective norepinephrine (NE) reuptake inhibitor that is used in humans for ADHD, inattention, and impulsivity associated with ADHD (237). These properties also make it a candidate therapy for psychostimulant addiction [for a review see Ref. (243)]. Like cocaine, ATO functions as a selective NE reuptake inhibitor that increases NE and dopamine in the prefrontal cortex (238, 239), but it does not have the abuse liability of other stimulants such as methylphenidate and desipramine (240).

##### Laboratory Animals

The relationship between ATO and impulsive behavior has been shown using several behavioral tasks in animals, such as the 5-CSRTT (76, 241), the SSRT task (76), and delay discounting (76, 235), but in other studies, ATO did not modify impulsivity (70, 241). In animal models of addictive behavior, ATO treatment was not effective at reducing cocaine self-administration in rats (71, 242–244). However, in combination with wheel running (245), ATO reduced cue-primed cocaine seeking in rats. It also reduced the strength of conditioned stimuli associated with nicotine in rats (246), attenuated nicotine withdrawal symptoms in mice (247), and reduced impulsive responding for i.v. cocaine in female rats (Smethells et al., under review). In our series of animal studies, we have modeled the combination approach with animals using some of the novel treatments described above. For instance, when combined with physical exercise ATO attenuated cocaine extinction, and cocaine-primed reinstatement in females but not in males (245). In a recent study, ATO was studied in rats responding for i.v. cocaine under a delay-discounting schedule with a small amount of cocaine available immediately, or a larger amount after a delay, treatment with ATO or ATO combined with PRO shifted the choice from the impulsive choice of a smaller-immediate cocaine delivery to the less impulsive choice of a larger-delayed cocaine delivery (248). However, the combined ATO–PRO treatment did not reduce impulsive cocaine seeking any further than either treatment alone. These animal studies suggest that ATO may be an effective treatment for psychostimulant addiction and for reducing impulsive behavior that underlies drug seeking.

##### Humans

Little data are available from human studies to confirm the potential for ATO to treat drug cocaine or other stimulant addiction. Some clinical investigations have not demonstrated a therapeutic effect of ATO on cocaine use (243, 244) or on the subjective effects of METH (249). However, Sofuoglu and Mooney (250) reported that ATO attenuated physiological and subjective effects of d-amphetamine. Others have shown fewer days of heavy alcohol drinking, less alcohol craving with ATO and longer abstinence from alcohol use with ATO treatment than with counseling by itself (251, 252).

#### Progesterone

Progesterone is used therapeutically in humans and for other primates for contraception, endometriosis, and maintaining pregnancies. It has also been shown in animal studies to indicate impulsive drug seeking and anxiety-like behaviors (248, 253, 254). PRO plays an important role in reducing drug seeking in rats [for review, see Ref. (6)], monkeys (255–257), and humans [for review, see Ref. (8)].

##### Laboratory Animals

In preclinical models, exogenously administered PRO and its primary metabolite, ALLO, attenuated acquisition, escalation of cocaine self-administration, and cocaine-primed reinstatement (54, 258) of cocaine seeking in rats (259, 260). Sex differences in the effects of ALLO have been reported with METH-primed reinstatement (64), and reinstatement was significantly reduced in female rats when they were treated with ALLO. However, ALLO had no effect on male rats [see Ref. (6) for a complete review]. In rats self-administering cocaine, concurrent running-wheel access was combined with PRO treatment, and the combination reduced extinction responding and cocaine-primed reinstatement in females but not males (216). However, in treatment-resistant males, the wheel access and PRO combination were more effective than wheel access or PRO alone. Studies of the effects of PRO on the rewarding effects of drugs show that rhesus monkeys maintained higher breakpoints for cocaine during the follicular than the luteal phase [Ref. (255); lowest dose only]. Also, rats self-administered more cocaine during the estrus phase of the estrous cycle, when estrogen levels are rising, than during proestrous, when PRO is relatively high (51, 259, 261–264).

##### Humans

In humans, during the follicular phase, when estrogen peaks, women report that cocaine is subjectively more rewarding than during the luteal phase, when PRO levels are have peaked [Ref. (265–267); see also Ref. (268)]. Human laboratory studies also indicate that PRO has an important role in nicotine addiction. For example, in a study of sensitivity to alcohol in women with premenstrual dysphoric disorder (PMDD), women reported a blunted physiological response and less intoxication after an alcohol infusion in the late luteal phase (high P/E) compared to the mid-follicular phase (low P/E) of the menstrual cycle indicating that PRO reduced the intoxicating effects of alcohol (269). In a recent study with both men and women, the effects of i.v. nicotine were assessed as a function of sex and menstrual cycle phase, and men reported greater subjective reactivity to nicotine, but women showed more physiological reactions (39). In women, this effect was diminished during the luteal phase (higher P/E ratio) compared to the follicular phase of the cycle. Women reported less nicotine reactivity, fewer negative symptoms, and better task performance during the luteal compared to the follicular phase suggesting that a higher P/E ratio may have alleviated nicotine’s negative effects. However, this sex difference finding was inconsistent with previous studies of the same i.v. nicotine infusion (250), oral intake (270), intranasal (271), and transdermal (272) nicotine administration, although phase of menstrual cycle was not a factor in these studies. The finding of greater subjective nicotine sensitivity in men vs. women was consistent with previous reports using intranasal nicotine (273) and smoked cocaine. Physiological findings of nicotine administration were consistent with the heart rate response (272) and diastolic blood pressure seen by others (274), but not with studies of nicotine and heart rate or blood pressure (250, 275).

In a recent smoking treatment study with either varenicline vs. placebo or nicotine patch vs. placebo patch in women, PRO levels were measured and compared to treatment outcome. This was the first study to identify a relationship between increasing levels of PRO and better abstinence outcomes in freely cycling women (236). The additive effect of rising PRO levels and treatment success was mainly found with the nicotine patch (vs. varenicline). There was a 23% increase in the odds of being abstinent within each of the 4 weeks of treatment in the luteal (PRO) + patch group. Based on animal research findings, clinical and preclinical researchers have examined the effects of exogenously administered PRO as a treatment for cocaine abuse. Comparable findings were obtained in humans who were treated with PRO. They showed reduced physiological and subjective rewarding effects of cocaine or cue-induced cocaine craving (8, 272, 276–279). Also, in clinical trials, PRO treatment reduced cocaine use in post-partum women in (280). Overall, there is strong accumulating evidence in human and animal studies, suggesting that, at least in females, PRO may serve as an efficacious pharmacological intervention for nicotine and cocaine addiction.

#### Treatment Combinations

Human studies suggest that combined therapies produce additive reductions in drug addiction compared to single treatment, and effects may vary with individual differences, such as male vs. female. For example, a review of 280 treatment studies for substance abuse disorders in men and women, published between 1975 and 2005, revealed better treatment outcomes for women than men (174). However, recent analysis of the multi-site COMBINE project, including 1383 men and women, reported that while there were sex differences in those seeking treatment for alcoholism and in those reporting alcohol treatment, there were no sex differences in the combined behavioral + naltrexone intervention, and the combination did not produce a better outcome than the individual treatments. Furthermore, women responded to naltrexone treatment and naltrexone + the control condition, medical management, similar to men (175). A recent review of combined pharmacotherapies (vs. single) for stimulant use disorder provided little evidence for an advantage of combined vs. monotherapies (281). Thus, further clinical work is needed with combined behavioral and pharmacological treatments for stimulant addiction to extend the promising results with laboratory animals to humans.

A recent study in rhesus monkeys showed reduced oral cocaine self-administration in female rhesus monkeys during the luteal phase of the menstrual cycle when PRO peaks compared with the follicular phase when estrogen peaks (Carroll et al., under review). In this study, monkeys received SACC concurrently with access to cocaine (0.4 mg/ml) under FR 4 schedules, and cocaine intake (milligram per kilogram) was compared in males and females during the follicular vs. the luteal phase of the menstrual cycle. When concurrent water was available with cocaine, females in the follicular phase consumed more cocaine than luteal females or males, an effect attributed to the lower PRO levels. Treatment with concurrent access to SACC along with cocaine resulted in reduced cocaine intake in both males and in females in both their follicular and luteal phases. An additive effect of PRO and SACC may have been occluded by a floor effect of SACC. However, a comparison of females across phases indicated a reduction in cocaine intake due to higher PRO (luteal phase) and to the additive effectiveness of PRO and SACC.

## Summary/Conclusion

Sex differences in behavioral dyscontrol were discussed in relation to drug addiction, as well as other factors that interact with sex differences to influence addictive behavior, such as impulsivity, compulsive binge intake of sweet substances, and age (adolescence). Each of these vulnerability factors has a substantial influence on behavioral dyscontrol and drug addiction. Not only do these individual differences explain the propensity for addiction in some individuals and not others but they also can be additive, presenting serious challenges to prevention and treatment once drug addiction has developed. Furthermore, these factors explain the propensity for addiction in some individuals and not others, which is instructive for designing prevention and treatment strategies. In addition, recent findings suggest that drug-prone individuals vs. those that are less sensitive to the aversive effects of drugs, further enhancing their vulnerability to addiction. Challenges in designing treatment for individuals with these addiction-prone characteristics are addressed by proposing novel treatments that take into account impulsive behavior and other forms of behavioral dyscontrol, such as excessive reward seeking, as well as sex, and hormonal conditions. Promising treatment strategies include behavioral manipulations, such as environmental enrichment (social and physical), such as exercise, or brief exposure to negative environmental consequences (e.g., punishment), and targeting individual differences with medications repurposed to address specific vulnerability factors, such as hormonal status (PRO), anxiety, or impulsivity (ATO), and combined behavioral and pharmacological therapies. Overall, the present review emphasizes that sex differences are intertwined with other major personality (or age-related) differences (e.g., anxiety, impulsivity, compulsive binging), and these factors are inseparable from sex differences when considering promising treatments for addiction. Thus, when considering development of treatments for drug abuse, sex is important, but other factors, such as a tendency for compulsive or impulsive behavior and anxiety, are equally as influential. Initial preclinical work has indicated that targeting these personality factors and sex differences has resulted in successful treatments for addiction.

## Conflict of Interest Statement

The authors declare that the research was conducted in the absence of any commercial or financial relationships that could be construed as a potential conflict of interest.
